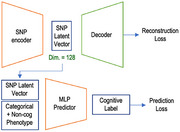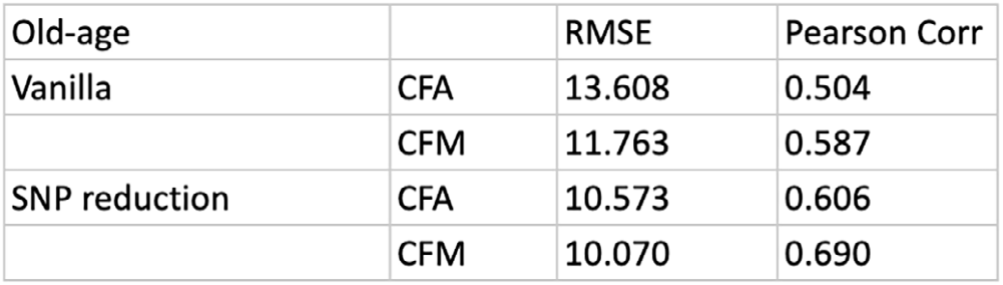# Integrating SNP Dimensionality Reduction and Bootstrapped k‐NN Imputation for Cognitive Function Prediction in AD‐BXD Mice

**DOI:** 10.1002/alz70855_100708

**Published:** 2025-12-23

**Authors:** Doyeong Hwang, Kyungwook Lee, Sungjoon Park, Soorin Yim, Kiyoung Kim, Amy R Dunn, Daniel M Gatti, Elissa J Chesler, Kristen MS O'Connell

**Affiliations:** ^1^ LG AI Research, Gangseo‐gu, Seoul, Korea, Republic of (South); ^2^ The Jackson Laboratory, Bar Harbor, ME, USA

## Abstract

**Background:**

The AD‐BXD mouse panel consists of genetically diverse lines that carry human familial Alzheimer's disease (FAD) transgenes, making it a valuable model for studying cognitive function and genetic factors associated with Alzheimer's disease (AD). The dataset includes single‐nucleotide polymorphism (SNP) data, cognitive phenotypes, and non‐cognitive phenotypes such as metabolic and sensory‐motor features. Cognitive phenotypes such as contextual fear acquisition (CFA; learning of fear‐related stimuli) and contextual fear memory (CFM; recall of fear‐related stimuli) are key measures of cognitive function. Challenges include sparse non‐cognitive features, requiring imputation, and the high dimensionality of SNP data relative to the limited sample size, necessitating robust dimensionality reduction. Our goal is to build a cognitive phenotype prediction model, and find biomarkers based on feature importance analysis with the model, despite above challenges.

**Method:**

We developed a predictive model for cognitive function using data generated from AD‐BXD mice at the Jackson Laboratory. The model uses SNP and non‐cognitive phenotype features before 7 months as input, and targets cognitive phenotype after 14 months. Step 1, we applied bootstrapped k‐NN imputation to address missing non‐cognitive data. Step 2, an autoencoder was trained to reduce SNP dimensionality by encoding essential reconstruction information. The latent vectors from the autoencoder were concatenated with imputed non‐cognitive features. Step 3, a multi‐layer perceptron (MLP) model was trained to predict cognitive labels, optimizing both the encoder and predictor iteratively to minimize mean squared error between predictions and ground truth. Step 2 and 3 are iterated.

**Result:**

Bootstrapped k‐NN imputation with non‐cognitive phenotypes produced 300 instances. MLP predictor using all features concatenated produced PCC(Pearson Correlation Coefficient) of 0.587 for CFM and 0.504 for CFA as predictive performance. SNP dimension produced PCC 0.69 for CFM and 0.606 for CFA. The feature importance analysis revealed biomarkers that are consistent with previously reported findings in the literature.

**Conclusion:**

The proposed model effectively utilized the genetically diverse AD‐BXD population for cognitive function prediction. Bootstrapped k‐NN imputation addressed missing data challenges, and SNP dimensionality reduction significantly enhanced predictive performance. Findings from feature importance analysis demonstrate the potential for identifying meaningful patterns and biomarkers to further AD‐related research.